# Scale dependent drivers of wild bee diversity in tropical heterogeneous agricultural landscapes

**DOI:** 10.1002/ece3.2360

**Published:** 2016-09-09

**Authors:** Parthiba Basu, Arpan Kumar Parui, Soumik Chatterjee, Aditi Dutta, Pushan Chakraborty, Stuart Roberts, Barbara Smith

**Affiliations:** ^1^ Centre for Pollination Studies University of Calcutta 35, B.C. Road Kolkata 700019 India; ^2^ 1 Waterloo Road Salisbury Wiltshire SP1 2JR UK; ^3^ Game and Wildlife Conservation Trust Burgate Manor Fordingbridge Hampshire SP6 1EF UK; ^4^ Centre for Agroecology Water and Resilience Coventry University Wolston Lane Coventry CV8 3LG UK

**Keywords:** Agricultural intensification, diversity, India, pesticide, scale, seminatural habitats, Wild bees

## Abstract

Factors associated with agricultural intensification, for example, loss of seminatural vegetation and pesticide use has been shown to adversely affect the bee community. These factors may impact the bee community differently at different landscape scales. The scale dependency is expected to be more pronounced in heterogeneous landscapes. However, the scale‐dependent response of the bee community to drivers of its decline is relatively understudied, especially in the tropics where the agricultural landscape is often heterogeneous. This study looked at effects of agricultural intensification on bee diversity at patch and landscape scales in a tropical agricultural landscape. Wild bees were sampled using 12 permanent pan trap stations. Patch and landscape characteristics were measured within a 100 m (patch scale) and a 500 m (landscape scale) radius of pan trap stations. Information on pesticide input was obtained from farmer surveys. Data on vegetation cover, productivity, and percentage of agricultural and fallow land (FL) were collected using satellite imagery. Intensive areas in a bee‐site network were less specialized in terms of resources to attract rare bee species while the less intensive areas, which supported more rare species, were more vulnerable to disturbance. A combination of patch quality and diversity as well as pesticide use regulates species diversity at the landscape scale (500 m), whereas pesticide quantity drove diversity at the patch scale (100 m). At the landscape scale, specialization of each site in terms of resources for bees increased with increasing patch diversity and FL while at the patch scale specialization declined with increased pesticide use. Bee functional groups responded differentially to landscape characteristics as well as pesticide use. Wood nesting bees were negatively affected by the number of pesticides used but other bee functional groups were not sensitive to pesticides. *Synthesis and Applications*: Different factors affect wild bee diversity at the scale of landscape and patch in heterogeneous tropical agricultural systems. The differential response of bee functional groups to agricultural intensification underpins the need for guild‐specific management strategies for wild bee conservation. Less intensively farmed areas support more rare species and are vulnerable to disturbance; consequently, these areas should be prioritized for conservation to maintain heterogeneity in the landscape. It is important to conserve and restore seminatural habitats to maintain complexity in the landscapes through participatory processes and to regulate synthetic chemical pesticides in farm operations to conserve the species and functional diversity of wild bees.

## Introduction

Agricultural intensification in past decades has led to large‐scale losses of farmland biodiversity (Robinson and Sutherland 2002; Le Feon et al. 2010) including wild bees that provide a critical ecosystem service, that is, pollination (R2Q14) (Hendrickx et al. 2007; Le Feon et al. 2010; Nicholls and Altieri 2012). The status of wild bee populations in agricultural landscapes is therefore a serious global conservation issue of recent times (Allen‐Wardell et al. 1998; Biesmeijer et al. 2006; Klein et al. 2007; 2013; Johnson et al. 2010; Potts et al. 2010; Nicholls and Altieri 2012; Becher et al. 2013). Factors related to agricultural intensification, for example, habitat fragmentation and loss, decreased landscape heterogeneity and increased pesticide usage, seem to be major causes for this decline (Steffan‐Dewenter and Tscharntke 2000; Ricketts 2001; Fahrig 2003; Desneux et al. 2007; Brosi et al. 2008; Calvillo et al. 2010; Geiger et al. 2010; Henry et al. 2012; Chakrabarti et al. 2014; Retschnig et al. 2015).

Insect abundance and richness in agricultural areas are affected by landscape characteristics at both the patch and the landscape levels (Gonthier et al. 2014). However, changes in bee community responses to different habitat characteristics due to fragmentation at different landscape scales are relatively understudied (Kevan 1999; Cane 2001; Steffan‐Dewenter 2002, 2003; Aizen and Feinsinger 2003; Calvillo et al. 2010; Kennedy et al. 2013). Increasing landscape scale heterogeneity may improve bee abundance and richness even in landscapes with little natural habitat (Kennedy et al. 2013). Different components of agricultural intensification might influence the community structure and interaction differently at different landscape scales (Tscharntke et al. 2012). In contrast to the more homogeneous farm landscapes in the northern hemisphere, landscape level heterogeneity is high in most tropical countries where the agricultural landscape is comprised of small landholdings and greater crop diversity. The binary comparisons (e.g., seminatural habitat vs. intensive cropping) conducted in the global north rarely account for the range of complexities associated with varying qualities of different habitats (Winfree et al. 2007; Kennedy et al. 2013). Despite the fact that India, along with a number of countries in the southern hemisphere, has undergone large‐scale agricultural intensification over the past decades (Oldroyd and Nanork 2009), the agricultural landscapes in most parts of the country are still heterogeneous mosaics of small farm land holdings and seminatural habitats. However, practices associated with agricultural intensification, such as widespread use of chemical inputs and the concomitant pressure on seminatural habitats, are still likely to have a negative impact. This makes India an ideal location for assessing the responses of wild bees to agricultural intensification in heterogeneous landscapes with varying patch characteristics.

We investigated how various landscape variables at different landscape scales in a heterogeneous vegetable farming area affected the diversity and vulnerability of the wild bee community. We hypothesized that the bee community will be influenced by agronomic inputs (we focused on pesticides) and the quality, complexity and extent of seminatural habitat. In view of this, we specifically hypothesized that:

- Bee diversity will respond to pesticide use and habitat characteristics such as patch productivity (representing habitat quality), patch diversity (PD) (representing habitat complexity), extent of land under cultivation and extent of land under fallow land (FL).
- Bee functional groups will respond differentially to pesticide use and habitat characteristics (productivity, PD, extent of land under cultivation, and FL).
- The important drivers will differ at the patch (100 m radii) and landscape scale (500 m radii).

## Materials and Methods

### Study site

This study was carried out in the northeastern Indian state of Tripura. The state shares its boundaries with Bangladesh on three sides (Majumder et al. 2012) and is an integral part of the Indo‐Burma biodiversity hot spot (Myers et al. 2000). The study region falls in the Khowai and Howrah river basin (35°32′229.68″E; 26°40′075.34″N to 36°32′11.98″E; 26°40″075.34″N) where the climate is characterized by a dry winter (November to February), moist summer (March to June), and monsoon (July to October). Monthly average temperatures range between 10 and 35°C and average annual rainfall is 2097 mm (Majumder et al. 2012).

We established a total of 12 study plots (in and around vegetable crop fields) of 200 × 200 m each. Average distance between the sites was 10 ± 1.25 km. Four of the plots were in areas of low agricultural intensification (hereafter referred to as the “low node”), three in areas of intermediate intensification (“mid node”), and five were in areas of high agricultural intensification (“high node”). All the plots were in the Teliamura subdivision in the Khowai and Jirania subdivision of West Tripura which has a heterogeneous landscape with a mosaic of agricultural lands interspersed with forested areas. The three classification of agricultural intensity was based on vegetation cover (NDVI, normalized difference vegetation index) (100 and 500 m spatial resolution) and pesticide usage (500 m spatial resolution) (see below for the detailed methodology). The low node sites were close to forested areas and were characterized by a high percentage of natural vegetation and low pesticide usage. The high node sites were comprised of large cropped areas with high pesticide usage and little natural vegetation cover. The intermediate sites (mid) included small cropped areas at the forest fringe, with both percentage of vegetation and pesticide usage at levels in between those found in the intensive and extensive areas. NDVI varied significantly along the intensification gradient at both 500 m (Kruskal–wallis ANOVA, *H* = 6.82, *P* = 0.03) and 100 m (Kruskal–wallis ANOVA, *H* = 6.77, *P* = 0.03) spatial resolution (ESM 1). However, pairwise comparisons showed the significant difference was between low and high nodes at both 500 m (multiple comparison between mean rank, *z* = 2.50, *P* = 0.04) and 100 m (multiple comparison between mean rank, *z* = 2.50, *P* = 0.03) spatial resolution.

The area under agriculture (AG) varied significantly along the intensification gradient at both 500 m (Kruskal–wallis ANOVA, *H* = 9.72, *P* = 0.01) and 100 m (Kruskal–wallis ANOVA, *H* = 8.69, *P* = 0.01) spatial resolution (ESM 1). Again, pairwise comparisons showed that the AG only varied significantly between low and high nodes at both 500 m (multiple comparison between mean rank, *z* = 3.10, *P* = 0.005) and 100 m (multiple comparison between mean rank, *z* = 2.91, *P* = 0.01) spatial resolution.

Pesticide input per acre (PIac) (see below for the detail methodology) varied significantly along the gradient (Kruskal–wallis ANOVA, *H* = 7.39, *P* = 0.02) (ESM 1), although PIac only varied between low and high nodes (multiple comparison between mean rank, *z* = 2.44, *P* = 0.04). Number of pesticide used (NOP) (see below for the detail methodology) also varied significantly along the intensification gradient (Kruskal–wallis ANOVA, *H* = 9.02, *P* = 0.01) (ESM 1). Again, the significant difference was only between low and high nodes (multiple comparison between mean rank, *z* = 2.98, *P* = 0.01).

### Bee sampling

Sampling was carried out from October 2012 to April 2013 on a monthly basis at each of the 12 sampling locations using pan traps. We followed Cane et al. (2000) and placed the pan traps of different colors at the same height as the floral resources in order to minimize the sampling bias. Although pan trapping does not capture all bee species during the flowering period, it has been reported as an efficient method that can provide insight into bee diversity that is otherwise unobtainable (Cane et al. 2000).

**Figure 1:**
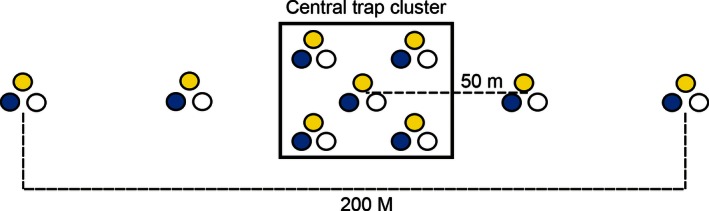
Schematic diagram of a trap cluster with additional traps on the wings in a given sampling location. Colored circles represent, respectively, colored pan traps.

A “pan trap station” was established at each location (Fig. 1). A cluster of five traps (each “trap” comprising three bowls, one each of white, blue, and yellow painted with UV reflective paints) (BOSNY paint) were set up within a randomly chosen vegetable field. Additional sets of two traps were placed on each side of the trap cluster at a distance of 50 m from the centre of the cluster, so that the pan trap station covered a total length of 200 m. Traps were filled with water and approximately 5 mg of washing powder was added to lower the surface tension (Fig. 1). Traps were left open for 24 h on each occasion. A total of 27 bowls were placed at each of the 12 sampling locations in each month of the study period. Therefore, a total of 2268 bowls were placed across 12 sampling locations over the period of 7 months. The average distance between the pan trap stations was 10 ± 1.25 km.

Coordinates of the pan trap stations were taken using GPS (Garmin, e‐treks‐30). The insects collected were at first rinsed with distilled water and then preserved in 70% alcohol in the field. In the laboratory, the bee specimens were identified to finest possible taxonomic resolution (usually genus and where possible, species) according to the couplet keys provided by Michener (2007) and with assistance from international taxonomic experts.

### Bee functional groups

Bees were classified by functional groups based on their nesting preferences. Information on bee nesting preferences was obtained from Michener (2007) and from our own observations. Bees in the genera *Amegilla, Andrena, Anthophora, Curvinomia, Halictus, Hylaeus,* and *Nomia* were grouped under soil nesting bee functional group (SOIL). The genera *Braunsapis* and *Ceratina* were categorized as tree–twig nesting functional group (TTWIG). Tree–twig nesting species generally nest in small holes in trees as well as tree–twigs and plant galls. The genera *Lithurgus, Trigona,* and *Xylocopa* were grouped under wood nesting functional group (WOOD). Wood nesting species generally nest in large trees and dead logs. The genera *Apis* and *Megachile* were not captured in any great quantity in the pan traps and were not included in any functional groups. The genus *Apis* was excluded from all the analyses except in the bee‐site network as it is well known that pan traps are not effective for sampling *Apis* (Cain et al. 2000; Brittain et al. 2010, 2013).

### Landscape parameters

To characterize the landscape, we estimated landscape composition from 30 m resolution Landsat TM imagery and the accuracy of our land use classification was estimated by a ground truthing (500 m radius) exercise at field‐based observations using a subsample of five randomly chosen sites. The AG, FL (barren land with no vegetation except as in some cases with grasses and small herbs), and PD were measured at two spatial resolutions of 100 and 500 m radius centered on the pan trap cluster. The NDVI was also measured at the same scales. NDVI was calculated as follows:

$$\text{NDVI}=\frac{\text{NIR}-\text{VIS}}{\text{NIR}+\text{VIS}}$$

where NIR = near infrared spectral reflectance and VIS = visible (red) spectral reflectance (Wu et al. 2010). NDVI provides an effective measure of photo‐synthetically active biomass (Tucker and Sellers 1986), and at a small spatial scale, it denotes the net primary productivity (Wu et al. 2014). All the landscape classification and analysis was performed by Arc GIS. Fragstat (v.2) software (http://www.umass.edu/landeco/research/fragstats/fragstats.html) was used to quantify PD.

### Farmer survey

To determine the pesticide input in each site, three farmers who cultivated crops within a 500 m radius of each pan trap station were randomly selected to take part in a survey. In the low and mid nodes, three selected farmers also happened to own all the lands within 500 m radius of the pan trap stations. However, in the high nodes and some sites in mid node, the three farmers were selected from a group of 5–6 farmers who owned all the lands within 500 m radius of the pan trap stations. The farmers provided information on the types of pesticides they used and estimated their total investment in pesticide (acre^−1^·year^−1^) during the survey period. The cost of pesticide (Rs.·mL^−1^) was similar within, and between, the 12 sites as was the cost of different pesticides. Therefore, pesticide investment per acre was used as an indication of amount of pesticide used. Information from the three farmers from each site was averaged and used for the analysis.

### Data analyses

The relationship between bee diversity (the response variable) and the NDVI, AG, area under FL, PD, pesticide investment per acre (PIac), and number of pesticides used (NOP) (the predictor variables) at two spatial scales (500 and 100 m) was analyzed using a linear regression model. Bee diversity was estimated using the Shannon Wiener index (Magurran 2003)

$$\text{BD}=-\sum_{i=1}^{s}p_{i}\cdot \ln p_{i}$$

where *S* = number of bee species, *p*_*i*_ = relative proportion of bee species for every *i*th category, ln = natural logarithm (base *e*).

All the data collected (diversity, as well as landscape variables) from, and around, each pan trap station were arithmetically averaged before analyses. The effect of combining predictor variables (all the subsets of predictor variables including the null model) was explored, and the best model was selected according to their lowest.

Akaike's information criterion corrected for small sample size (AICc) value (∆AICc > 2) (Burnham and Anderson 2002). The effect of landscape and pesticide variables on bee functional group was also investigated; the abundance of each functional group was tested separately as a response variable. Normality and heteroscedasticity was checked by inspecting the Normal Q‐Q plots and standardized residuals vs. fitted plots from “R” output. Response variables were log transformed when necessary to achieve normality. Multicollinearity between predictor variables was checked using the variance inflation factor (VIF). VIF values >10 were considered as multicollinear (Kutner et al. 2004; Dormann et al. 2013). Analyses were performed using R statistical software (version 3.0.1) with “fmsb” and AICcmodavg packages.

A bee species‐site network was constructed to illustrate the mutualistic association between bee species and the specific habitats, here represented by a sampling site. A bee‐site network was calculated using the average number of bees trapped from 12 sites in a quantitative availability matrix, where sites were entered as rows and bee species were placed in columns. Different network parameters were calculated at both the network, as well as the species level. In our bee‐site network, species level refers to sites. The overall level of specialization (*H*′) was measured at the network level. $H_{2}^{\prime }$ was calculated as standardized two‐dimensional Shannon entropy. The values of $H_{2}^{\prime }$ ranged between “0” (complete generalization) and “1” (complete specialization). Specialization at site level (similar to species‐level analysis in a mutualistic network) was calculated as standardized Kullback‐Leibler distance (*d*′) of each site (a site in a bee‐site network is analogous to plant species in a bee–plant mutualistic network). The standardized specialization index (*d*′) at site level also ranges between “0” and “1,” indicating complete generalization and complete specialization, respectively. Nestedness of the network was calculated using the networking metric based on overlap and decreasing fill (NODF metric) (Almeida‐Neto et al. 2008). Recorded NODF values were compared with 100 random matrices generated by the null model. An NODF value close to “0” indicated the absence of nestedness and a value close to 100 indicated total nestedness. Network analyses were performed using “R” (version 3.0.1, https://www.r-project.org/) with “bipartite,” “ggplot2,” “igraph,” and “SNA” packages.

## Results

### Bee community

A total of 56 morpho‐species, from five families and 14 genera, were collected from the pan traps. The most abundant family was Halictidae comprising 34 morpho‐species from four genera. Andrenidae and Colletidae were two rare families with only one genus and one morpho‐species from each. *Lasioglossum* spp. (Halictidae) comprised the highest proportion of individuals (93.18% of the total catch) and species (46.43%).

Bee diversity was highest in the low node (*H*′ = 1.47 ± 0.12) when compared to the high node (*H*′ = 1.15 ± 0.06) (Mann–Whitney *U*‐test, *Z*_adjusted_ = 2.33, *P*_adjusted_ = 0.02) but was not significantly different from the mid node (*H*′ = 1.08 ± 0.16) (Mann–Whitney *U*‐test, *Z*_adjusted_ = 1.27, *P*_adjusted_ = 0.22). There was no difference between the mid and high nodes (Mann–Whitney *U*‐test, *Z*_adjusted_ = 0.29, *P*_adjusted_ = 0.76).

**Figure 2:**
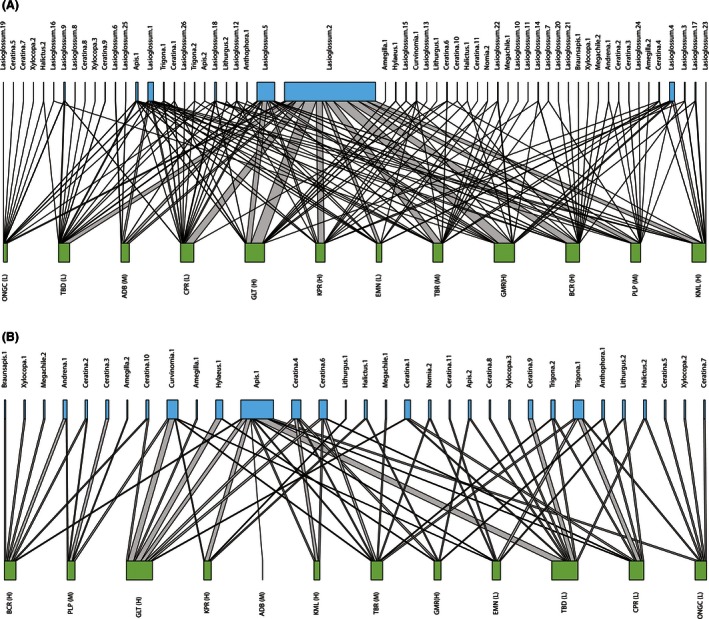
(A) Bee‐site network showing connectance between bee species and different sites. (B) Bee‐site network excluding *Lasioglossum* spp. from the bee community. Bee species are indicated by the upper boxes and sites are indicated by the boxes in the lower row. Box width corresponds to the relative fraction of interactions a bee species and different sites contribute to the network. Width of interaction lines is proportional to the number of observed interactions. L, low node; M, mid node and H, high node.

The bee species‐site network with all the bee species is shown in Figure 2A. Network level nestedness (NODF) and specialization ($H_{2}^{\prime }$) were 19.53 and 0.28, respectively. However, nestedness decreased (10.72) and specialization (0.58) increased considerably when we removed *Lasioglossum* spp. from the network (Fig. 2B). The site level network (which is analogous to the species‐level network in a mutualistic interaction network where network matrices are calculated on the basis of lower level taxa and not the whole network) with respect to high node sites showed relatively less specialization (*d*′ = 0.06 ± 0.01) when compared with the low (*d*′ = 0.12 ± 0.03) and mid nodes (*d*′ = 0.12 ± 0.06). The same analysis, after removing *Lasioglossum* from the network, showed comparatively lower specialization in low nodes (0.04 ± 0.02) compared to mid (0.17 ± 0.17) and high nodes (0.14 ± 0.09).

### Functional groups

The abundance of soil nesters and wood nesters varied significantly across the nodes (*F*_2,9_ = 4.55, *P* = 0. 04, *F*_2,9_ = 6.27, *P* = 0.02, respectively). Soil nesters were significantly more abundant at the high node (*X* = 36.55 ± 6.28) when compared to the low node (*X* = 18.3 ± 5.17) (Tukey's HSD, *P* < 0.05) while wood nesters were significantly more abundant at the low node (*X* = 0.71 ± 0.18) when compared to the high node (*X* = 0.098 ± 0.06) (Tukey's HSD, *P* = 0.02). Abundance of the tree–twig nesters did not vary significantly across the nodes (*F*_2,9_ = 0.314, *P* = 0.738), and there was no significant difference between low (*X* = 0.675 ± 0.222), mid (*X* = 0.424 ± 0.212), and high node (*X* = 0.68 ± 0.242) (Tukey's HSD, *P* > 0.05 for all combinations).

### Scale‐specific relationship of bee species diversity with landscape and pesticide variables

Bee species diversity (*H*′) was best explained by a significant positive relationship with NDVI (*t* = 3.902, *P* = 0.008) and extent of FL (*t* = −3.640, *P* = 0.01), and a significantly negative relationship was found with both pesticide investment per acre (PIac) (*t* = −3.702, *P* = 0.01) and number of pesticides used (NOP) (*t* = −2.661, *P* = 0.04) at 500 m radii. The best model explained 62% of the variation (Table 1). Although bee species diversity was poorly explained by the parameters used at 100 m radii (the best model explained 27% of the variation), there was a significant negative relationship with pesticide investment per acre (PIac) (*t* = −2.279, *P* = 0.04) (Table 2).

**Table 1:** Effects of different landscape and pesticide variables on bee community, bee functional groups, and site specialization around 500 m radius of pan trap station. Only the best combined models are described here

Response (500 m radii)	Best model	AIC	AICc
Bee diversity (BD)	**NDVI** + **PIac** + **FL** + **NOP** + PD	−5.39	26.61
Site specialization index (HSI)	**PD+ FL**	26.59	32.31
Soil nesters (SOIL)	**FL** + **NOP** + **PD** + **PIac**	9.75	26.54
Tree–twig nesters (TTWIG)	PIac	13.48	16.48
Wood nesters (WOOD)	**NOP** + PD	57.74	63.45

PD, patch diversity; PIac, pesticide investment; NOP, number of pesticides; NDVI, normalized difference vegetation index; AG, area under agriculture; FL, area under fallow; AIC, Akaike's information criteria.

Bold variables indicate significant relationship.

**Table 2:** Effects of landscape and pesticide variables bee community, bee functional groups, and site specialization around 100 m radius of pan trap station. Only the best combine models are described here

Response (100 m radii)	Best model	AIC	AICc
Bee diversity (BD)	**PIac +** FL	−3.98	1.73
Site specialization index (HSI)	**PIac** + FL	22.77	28.48
Soil nesters (SOIL)	**FL** + **NOP** + **PIac** + **PD**	3.65	20.45
Tree–twig nesters (TTWIG)	NDVI + NOP	59.67	65.39
Wood nesters (WOOD)	**NOP** + PD	57.74	63.45

PD, patch diversity; PIac, pesticide investment; NOP, number of pesticides; NDVI, normalized difference vegetation index; AG, area under agriculture; FL, area under fallow; AIC, Akaike's information criteria.

Bold variables indicate significant relationship.

### Scale‐specific relationships of site specialization with landscape and pesticide variables

Site specialization was best explained (39% of variation explained) by positive relationships with PD (*t* = 2.40, *P* = 0.04) and FL (*t* = 2.350, *P* = 0.04) within 500 m radii (Table 1); meaning a site was more specialized in terms of resource provision for bees with higher PD and extent of FL at the landscape scale. At 100 m radius site, specialization was explained by a negative relationship with pesticide investment per acre PIac (*t* = −3.236, *P* = 0.01) where the best model explained 55% variation (Table 2).

### Scale‐specific relationship of the abundances of different bee functional groups with landscape and pesticide variables

With respect to soil nesting bees, the best model at 500 m radii explained 69% of the variation, (Table 1) and 100 m radii it explained 81% of the variation (Table 2). The model (R2Q81) included a negative relationship with FL (*t* = −2.926, *P* = 0.02, *t* = −4.340, *P* = 0.003, respectively) and PD (*t* = −2.532, *P* = 0.039 and *t* = −2.418, *P* = 0.046, respectively). The model showed a significant positive relationship with NOP (*t* = 2.595, *P* = 0.035 and *t* = 3.898, *P* = 0.005, respectively) and PIac (*t* = 2.515, *P* = 0.04 and *t* = 2.574, *P* = 0.036, respectively). Wood nesting bees were negatively correlated with the number of pesticides (NOP) at both 500 m (*t* = −2.595, *P* = 0.035) and 100 m radii (*t* = −3.502, *P* = 0.006) where both the best models explained 51% of the variation. For tree and twig nesting species, no significant relationships were established with any of the predictor variables (Table 2).

## Discussion

Among these factors associated with agricultural intensification, the loss of natural vegetation in the agricultural landscape (Knight et al. 2009; Kennedy et al. 2013) and increasing insecticide use (Thompson 2001; Desneux et al. 2007) are reported as major drivers of decline in bee community, and this study therefore focused on these two important parameters. In our study, bee diversity was higher in the low agricultural intensification areas that are characterized by low pesticide use and relatively high proportion of seminatural habitats in the landscape. Interestingly, agricultural intensification seemed to favor soil nesting bees and they were found in higher numbers in high intensification areas that have a higher proportion of bare soil compared to the other two nodes. The soil nesting bees are dominated by the genus *Lasioglossum* that are small, non‐Apid wild bees (Michener 1974). Dominance of one particular group due to agricultural intensification is a well‐known phenomenon where increasing agricultural intensification and losses of quality patches may shift pollinator communities toward common and ubiquitous taxa (Carré et al. 2009; Kennedy et al. 2013). It may also be that our sampling method (i.e., pan trapping) is biased toward *Lasioglossum* spp. as they are readily attracted to these traps (Geroff et al. 2014). However, other functional groups, such as the wood nesters, were negatively affected by the number of pesticides used and occurred in relatively lower numbers in areas of high agricultural intensification. Tree and twig nesting species did not respond to any of the variables we tested.

Using network analysis, we investigated “nestedness” in a “bee‐site network.” We have considered a site as a habitat containing specific resources for the visiting bees. A highly nested network indicates a network that supports many connections, low competition, and increased coexistence where specialist species interact with the generalist species (Bastolla et al. 2009). A nested community will also be somewhat resilient to habitat loss (Fortuna and Bascompte 2006). Similar to bee species–plant species network, a bee species‐site network can be also viewed as a mutualistic association. In such an association, a given bee species exploits sites that offer specific resources for their survival. On the other hand, sites are also mutually benefited by the presence of the bee species that can influence ecosystem processes crucial for the habitat's sustainability. Analysis of our studied agricultural patches showed comparatively low nestedness in the network. Nestedness decreased when *Lasioglossum*s pp. were removed from the whole network making the network more vulnerable. Bees dependent on plants for shelter are much more vulnerable to loss of seminatural habitat (Carré et al. 2009; Vanbergen 2014). Intensive agricultural landscapes with sparse or no seminatural vegetation offer them little nesting opportunity. On the other hand, soil nesters can explore both intensive and nonintensive areas based on the availability of bare lands in each area and are therefore have more choice. Our observed bee‐site network therefore appears vulnerable to habitat loss and fragmentation for nonsoil nesting bees and underpins the importance of the generalist species such as *Lasioglossum* for maintaining a well‐connected bee‐site network.

Another measure of vulnerability is network specialization. A highly specialized network indicates reduced redundancy among interactions in the network and means that the network is sensitive to disturbance (Naeem and Li 1997; Yachi and Loreau 1999). Specialization increased considerably when the generalist *Lasioglossum* spp. were removed from the whole network. Again the role of *Lasioglossum* in maintaining a less specialized network (and therefore a more resilient one) was established in our study area. Our study demonstrates the higher vulnerability of low agricultural intensification areas compared with intensively farmed areas in the network with all bee species included. However, the presence of rarer bee species at the less intensive sites makes such sites more susceptible to disturbance compared to networks in more intensive areas dominated by generalist species, for example, *Lasioglossum*. As removal of *Lasioglossum* from the network makes the high and mid intensive sites more specialized, it indicates that the presence of *Lasioglossum* spp. also masks the degradation of the networks in such areas.

Patch level (small spatial scale) or landscape level (large spatial scale) selection has been observed in a number of taxa (Kruess 2003; Holland et al. 2004; Tscharntke et al. 2012). Insect abundance and richness in agricultural landscapes have been reported to be sensitive to both patch level and landscape level factors including pesticide and landscape complexity calling for a scale‐specific approach when designing conservation strategies (Gonthier et al. 2014). The present study also demonstrates this, as different variables were important at the patch level (100 m radius) and the landscape level (500 m radius).

NDVI provides an effective measure of photo‐synthetically active biomass (Tucker and Sellers 1986), and at a small spatial scale, it reflects the net primary productivity (Vogeler et al. 2014; Wu et al. 2014). Bee diversity was greater in areas with a higher NDVI and extent of FL. Species diversity was clearly scale sensitive in our study. Diversity was positively associated with both decreasing number and quantity of pesticides used at a large spatial scale. However, at a small spatial scale, only the number of pesticides used was significant. This suggests that low pesticide input and a heterogeneous landscape with both higher percentage of vegetation cover along with patches of FL at multiple spatial scales is important for maintaining bee diversity in heterogeneous agricultural farmlands in a country such as India.

Due to the low abundance of most of the species captured in the pan traps, we could not perform any species‐level analysis and were also unable to categorize all bees into functional groups. For example, *Apis dorsata* and *Apis cerana* are social species which were captured in very low numbers, but we do know from other studies that pan traps do not capture honeybees well. Soil nesting bees, mainly dominated by *Lasioglossum* spp., showed a preference for low PD and tended to be associated with higher number of pesticides used and investment in pesticides (a proxy measure of the quantity of pesticides used). These bees' abundance increased in landscapes where PD and FLs decreased and the amount of pesticide and the number of pesticides used increased. This was shown at both spatial scales. We therefore conclude that these conditions are most likely due to the increased availability of nesting opportunities in a homogenous habitat that are favorable for dominant soil nesting *Lassioglossum* species and more than pesticide use nesting site availability is perhaps more of a determining factor for the soil nesters. However, wood nesters were negatively affected by pesticide application (at both spatial scales). Therefore, instead of responding to landscape heterogeneity, the wood nesting bee community is generally affected by the number of pesticides used. A mixed‐model analyses of data sets from across the globe showed that above ground nesting bees are more affected by agricultural intensification and isolation from seminatural habitats (Williams et al. 2010). A meta‐analysis by Brittain and Potts (2011) also shows that above ground nesting bees are adversely affected in pesticide intensive landscapes.

This study indicates a need for specific conservation strategies at different scales. In heterogeneous tropical landscapes, as found in a country such as India, average landholding tends to be very small, often less than one acre. Therefore, at landscape scale (a 500 m radius), farmers would need to work together to ensure that there is sufficient habitat complexity to support pollinator populations. As less intensively farmed areas support rare species, they need to be prioritized for habitat conservation. Individually, on their own farms, farmers can protect pollinator populations by reducing the number and quantity of pesticides.

## Conflict of Interest

None declared.

## References

[ece32360-bib-0001] Aizen, M. A. , and P. Feinsinger . 2003 Bees not to be? Responses of insect pollinator faunas and flower pollination to habitat fragmentation Pp. 111–129 *in* Bradshaw G. A. and Marquet P. A., eds. How landscapes change: human disturbance and ecosystem fragmentation in the Americas. Springer‐Verlag, Berlin.

[ece32360-bib-0002] Allen‐Wardell, G. , R. Bitner , A. Burquez , S. Buchmann , J. Cane , P. Allen , et al. 1998 The potential consequences of pollinator declines on the conservation of biodiversity and stability of food crop yields. Conserv. Biol. 12:8–17.

[ece32360-bib-0003] Almeida‐Neto, M. , P. Guimarães , P. R. Jr Guimarães , R. D. Loyola , and W. Ulrich . 2008 A consistent metric for nestedness analysis in ecological systems: reconciling concept and measurement. Oikos 117:1227–1239.

[ece32360-bib-0004] Bastolla, U. , M. A. Fortuna , A. Pascual‐Garcia , et al. 2009 The architecture of mutualistic networks minimizes competition and increases biodiversity. Nature 458:1018–1020. 19396144 10.1038/nature07950

[ece32360-bib-0005] Becher, M. A. , J. L. Osborne , P. Thorbek , P. J. Kennedy , and V. Grimm . 2013 Towards a systems approach for understanding honeybee decline: a stocktaking and synthesis of existing models. J. Appl. Ecol. 50:868–888. 24223431 10.1111/1365-2664.12112 PMC3810709

[ece32360-bib-0006] Biesmeijer, J. C. , S. P. M. Roberts , M. Reemer , R. Ohlemüller , M. Edwards , T. Peeters , et al. 2006 Parallel declines in pollinators and insect‐pollinated plants in Britain and the Netherlands. Science 313:351–354. 16857940 10.1126/science.1127863

[ece32360-bib-0007] Brittain, C. , and S. G. Potts . 2011 The potential impacts of insecticides on the life‐history traits of bees andthe consequences for pollination. Basic Appl. Ecol. 12:321–333.

[ece32360-bib-0009] Brittain, C. , M. Vighi , R. Bommarco , J. Settele , and S. G. Potts . 2010 Impacts of a pesticide on pollinator species richness at different spatial scales. Basic Appl. Ecol. 11:106–115.

[ece32360-bib-0010] Brittain, C. , N. Williams , C. Kremen , and A. M. Klein . 2013 Synergistic effects of non‐Apis bees and honey bees for pollination services. Proc. Biol. Sci. 280:20122767. 23303545 10.1098/rspb.2012.2767 PMC3574329

[ece32360-bib-0011] Brosi, B. J. , G. C. Daily , T. M. Shih , F. Oviedo , and G. Duran . 2008 The effects of forest fragmentation on bee communities in tropical countryside. J. Appl. Ecol. 45:773–783.

[ece32360-bib-0012] Burnham, K. P. , and D. R. Anderson . 2002 Model selection and multimodal inference: a practical information‐theoretic approach, 2nd edn Springer, New York, NY.

[ece32360-bib-0013] Calvillo, L. M. , V. M. Ramírez , V. P. Tabla , and J. Navarra . 2010 Bee diversity in a fragmented landscape of the Mexican neotropic. J. Insect Conserv. 14:323–334.

[ece32360-bib-0014] Cane, J. H. 2001 Habitat fragmentation and native bees: a premature verdict? Ecol. Soc. Conserv. Ecol. 5:3.

[ece32360-bib-0015] Cane, J. H. , R. L. Minckley , and L. J. Kervin . 2000 Sampling bees (Hymenoptera: Apiformes) for pollinator community studies: pitfalls of pan‐trapping. J. Kansas Entomol. Soc. 74:225–231.

[ece32360-bib-0017] Carré, G. , P. Roche , R. Chifflet , N. Morison , R. Bommarco , J. Harrison‐Cripps , et al. 2009 Landscape context and habitat type as drivers of bee diversity in European annual crops. Agric. Ecosyst. Environ. 133:40–47.

[ece32360-bib-0018] Chakrabarti, P. , S. Rana , S. Sarkar , B. Smith , and P. Basu . 2014 Pesticide‐induced oxidative stress in laboratory and field populations of native honey bees along intensive agricultural landscapes in two Eastern Indian states. Apidologie 14:107–129.

[ece32360-bib-0019] Desneux, N. , A. Decourtye , and J. M. Delpuech . 2007 The sublethal effects of pesticides on beneficial arthropods. Annu. Rev. Entomol. 52:81–106. 16842032 10.1146/annurev.ento.52.110405.091440

[ece32360-bib-0501] Dormann, C. F. , J. Elith , S. Bacher , C. Buchmann , G Carl , G Carré , et al. 2013 Collinearity: a review of methods to deal with it and a simulation study evaluating their performance. Ecography 35:27–46.

[ece32360-bib-0020] Fahrig, L. 2003 Effects of habitat fragmentation on biodiversity. Annu. Rev. Ecol. Evol. Syst. 34:487–515.

[ece32360-bib-0021] Fortuna, M. A. , and J. Bascompte . 2006 Habitat loss and the structure of plant–animal mutualistic networks. Ecol. Lett. 9:281–286. 16958893 10.1111/j.1461-0248.2005.00868.x

[ece32360-bib-0022] Geiger, F. , J. Bengtsson , F. Berendse , W. W. Weisser , M. Emmerson , M. B. Morales , et al. 2010 Persistent negative effects of pesticides on biodiversity and biological control potential on European farmland. Basic Appl. Ecol. 11:97–105.

[ece32360-bib-0023] Geroff, R. K. , J. Gibbs , and K. W. McCravy . 2014 Assessing bee (Hymenoptera: Apoidea) diversity of an Illinois restored tallgrass prairie: methodology and conservation considerations. J. Insect Conserv. 18:951–964.

[ece32360-bib-0024] Gonthier, D. J. , K. K. Ennis , S. Farinas , H. Y. Hsieh , A. L. Iverson , P. Batáry , et al. 2014 Biodiversity conservation in agriculture requires a multi‐scale approach. Proc. Biol. Sci., 281:20141358. 25100703 10.1098/rspb.2014.1358 PMC4132690

[ece32360-bib-0026] Hendrickx, F. , J. P. Maelfait , W. Van Wingerden , O. Schweiger , M. Speelmans , S. Aviron , et al. 2007 How landscape structure, land‐use intensity and habitat diversity affect components of total arthropod diversity in agricultural landscapes. J. Appl. Ecol. 44:340–351.

[ece32360-bib-0027] Henry, M. , M. Begum , F. Requier , O. Rollin , JF. Odoux , P. Aupinel , et al. 2012 A common pesticide decrease foraging success and survival in honey bees. Science 336:348–350. 22461498 10.1126/science.1215039

[ece32360-bib-0028] Holland, J. D. , D. G. Bert , and L. Fahrig . 2004 Determining the spatial scale of species' response to habitat. Bioscience 54:229–235.

[ece32360-bib-0030] Johnson, R. M. , M. D. Ellis , C. Mullin , and M. Frazier . 2010 Pesticides and honey bee toxicity ‐ USA. Apidologie 41:312–331.

[ece32360-bib-0031] Kennedy, C. M. , E. Lonsdorf , M. C. Neel , N. M. Williams , T. H. Ricketts , R. Winfree , et al. 2013 A global quantitative synthesis of local and landscape effects on wild bee pollinators in agro ecosystems. Ecol. Lett. 16:584–599. 23489285 10.1111/ele.12082

[ece32360-bib-0032] Kevan, P. 1999 Pollinators as bioindicators of the state of the environment: species, activity and diversity. Agric. Ecosyst. Environ. 74:373–393.

[ece32360-bib-0033] Klein, A. M. , B. E. Vaissiere , J. H. Cane , I. Steffan‐Dewenter , S. A. Cunningham , C. Kremen , et al. 2007 Importance of pollinators in changing landscapes for world crops. Proc. Biol. Sci. 274:303–313. 17164193 10.1098/rspb.2006.3721 PMC1702377

[ece32360-bib-0034] Knight, M. E. , J. L. Osborne , R. A. Sanderson , R. J. Hale , A. P. Martin , and D. Goulson . 2009 Bumblebee nest density and the scale of available forage in arable landscapes. Insect Conserv. Divers. 2:116–124.

[ece32360-bib-0036] Kruess, A. 2003 Effects of landscape structure and habitat type on a plant‐herbivore‐parasitoid community. Ecography 26:283–290.

[ece32360-bib-0502] Kutner, M. H. , C. J. Nachtsheim , J. Neter , and W. Li . 2004 Applied linear statistical models. McGraw Hill Higher Education.

[ece32360-bib-0037] Le Feon, V. L. , A. Schermann‐Legionnet , Y. Delettre , S. Aviron , R. Billeter , R. Bugter , et al. 2010 Intensification of agriculture, landscape composition and wild bee communities: a large scale study in four European countries. Agric. Ecosyst. Environ. 137:143–150.

[ece32360-bib-0038] Magurran, A. E. 2003 Measuring biological diversity. Wiley Blackwell, Oxford, UK.

[ece32360-bib-0039] Majumder, J. P. , P. Bhattacharjee , K. Majumdar , C. Debnath , and B. K. Agarwala . 2012 Documentation of herpetofaunal species richness in Tripura, northeast India. NeBio 3:60–70.

[ece32360-bib-0040] Michener, C. D. 1974 The social behavior of the bees: a comparative study. Harvard Univ. Press, Cambridge, MA Pp. 404.

[ece32360-bib-0503] Michener, C. D. 2007 The bees of the world (2nd edition). Johns Hopkins Univ. Press Baltimore, MD.

[ece32360-bib-1001] Myers, N. , R. A. Mittermeier , C. G. Mittermeier , G. A. B. da Fonseca , and J. Kent . 2000 Biodiversity hotspots for conservation priorities. Nature. 403:853–858. 10706275 10.1038/35002501

[ece32360-bib-0041] Naeem, S. , and S. Li . 1997 Biodiversity enhances ecosystem reliability. Nature 390:507–509.

[ece32360-bib-0042] Nicholls, C. I. , and M. A. Altieri . 2012 Plant biodiversity enhances bees and other insect pollinators in agroecosystems. A review. Agron. Sustain. Dev. 33:257–274.

[ece32360-bib-0043] Oldroyd, B. P. , and P. Nanork . 2009 Conservation of Asian honey bees. Apidologie 40:296–312.

[ece32360-bib-0044] Potts, S. G. , J. C. Biesmeijer , C. Kremen , P. Neumann , O. Schweiger , and W. E. Kunin . 2010 Global pollinator declines: trends, impacts and drivers. Trends Ecol. Evol. 25:345–353. 20188434 10.1016/j.tree.2010.01.007

[ece32360-bib-0045] Retschnig, G. , G. R. Williams , R. Odemer , J. Boltin , C. D. Potto , M. M. Mehmann , et al. 2015 Effects, but no interactions, of ubiquitous pesticide and parasite stressors on honey bee (*Apismelifera*) lifespan and behaviour in a colony environment. Environ. Microbiol. 17:4322–4331. doi:10.1111/1462‐2920.12825. 25728008 10.1111/1462-2920.12825

[ece32360-bib-0046] Ricketts, T. H. 2001 The matrix matters: effective isolation in fragmented landscapes. Am. Nat. 158:87–99. 18707317 10.1086/320863

[ece32360-bib-0047] Robinson, R. A. , and W. J. Sutherland . 2002 Post‐war changes in arable farming and biodiversity in Great Britain. J. Appl. Ecol. 39:139–176.

[ece32360-bib-0048] Steffan‐Dewenter, I. 2002 Landscape context affects trap‐nesting bees, wasps, and their natural enemies. Ecol. Entomol. 27:631–637.

[ece32360-bib-0049] Steffan‐Dewenter, I. 2003 Importance of habitat area and landscape context for species richness of bees and wasps in fragmented orchard meadows. Conserv. Biol. 17:1036–1044.

[ece32360-bib-0050] Steffan‐Dewenter, I. , and T. Tscharntke . 2000 Butterfly community structure in fragmented habitats. Ecol. Lett. 3:449–456.

[ece32360-bib-0051] Thompson, H. M. 2001 Assessing the exposure and toxicity of pesticides to bumblebees (*Bombus* sp.). Apidologie 32:305–321.

[ece32360-bib-0052] Tscharntke, T. , J. M. Tylianakis , T. A. Rand , R. K. Didham , L. Fahrig , P. Batáry , et al. 2012 Landscape moderation of biodiversity patterns and processes ‐ eight hypotheses. Biol. Rev. 87:661–685. 22272640 10.1111/j.1469-185X.2011.00216.x

[ece32360-bib-0053] Tucker, C. J. , and P. Sellers . 1986 Satellite remote sensing of primary production. Int. J. Remote Sens. 7:1395–1416.

[ece32360-bib-0054] Vanbergen, A. J. 2014 Landscape alteration and habitat modification: impacts on plant pollinator systems. Curr. Opin. Insect Sci. 5:44–49: doi:10.1016/j.cois.2014.09.004. 10.1016/j.cois.2014.09.004 32846741

[ece32360-bib-0056] Vogeler, J. C. , T. Hudak , L. A. Vierlingc , J. Evans , P. Green , and K. T. Vierling . 2014 Terrain and vegetation structural influences on local avian species richness in two mixed‐conifer forests. Remote Sens. Environ. 147:13–22.

[ece32360-bib-0057] Williams, N. M. , E. E. Crone , T. H. Roulston , R. L. Minckley , L. Packer , and S. G. Potts . 2010 Ecological and life‐history traits predict bee species responses to environmental disturbances. Biol. Conserv. 143:2280–2291.

[ece32360-bib-0058] Winfree, R. , N. M. Williams , J. Dushoff , and C. Kremen . 2007 Native bees provide insurance against ongoing honey bee loss. Ecol. Lett. 10:1105–1113. 17877737 10.1111/j.1461-0248.2007.01110.x

[ece32360-bib-0059] Wu, W. , P. Yang , P. H. Tang , Q. Zhou , Z. Chen , and R. Shibasaki . 2010 Characterizing spatial patterns of phenology in cropland of China based on remotely sensed data. Agric. Sci. China 9:101–112.

[ece32360-bib-0060] Wu, X. , M. Lv , Z. Jin , R. Michishita , J. Chen , and H. Tian . 2014 Normalized difference vegetation index dynamic and spatiotemporal distribution of migratory birds in the Poyang Lake wetland, China. Ecol. Indic. 47:217–230.

[ece32360-bib-0061] Yachi, S. , and M. Loreau . 1999 Biodiversity and ecosystem productivity in a fluctuating environment: the insurance hypothesis. Proc. Natl Acad. Sci. USA 96:1463–1468. 9990046 10.1073/pnas.96.4.1463 PMC15485

